# Stress in Immature Incisor Treated With Regenerative Endodontics or Restored With Bulk‐Fill Resin Composite: A 2D Finite Element Analysis

**DOI:** 10.1111/aej.12981

**Published:** 2025-07-21

**Authors:** Gabriela Leite de Souza, Gabriel Felipe de Bragança, Andomar Bruno Fernandes Vilela, Airin Karelys Avendaño Rondón, Bill Kahler, Carlos José Soares, Camilla Christian Gomes Moura

**Affiliations:** ^1^ Department of Endodontics, School of Dentistry Federal University of Uberlândia Uberlândia Minas Gerais Brazil; ^2^ Department of Operative Dentistry and Dental Materials, School of Dentistry Federal University of Uberlândia Uberlândia Minas Gerais Brazil; ^3^ Department of Restorative and Reconstructive Dentistry, Sydney Dental School University of Sydney Sidney Australia

**Keywords:** apexification, finite element analysis, nonvital tooth, regenerative endodontics

## Abstract

This study investigated the stress distribution on teeth undergoing regenerative endodontic treatment (RET) using two‐dimensional finite element analysis (2D FEA). Five 2D‐FEA models of immature incisors were developed, involving immediately after RET, models after RET varying root length and thickness: 10% increase in root length and root thickness; 10% increase in root length and 20% increase in root dentin thickness; 10% increase in root length and 40% increase in root dentin thickness; and a model after MTA apexification reinforced with resin composite. A biting load of 100 N was applied, and modified von Mises stress was analysed. Increasing root dentin thickness after RET provides limited benefit in the stress distribution, mainly by reducing the stress‐to‐area ratio in dentin. MTA apexification followed by resin composite restoration decreased intraradicular stress at the cervical region compared to RET. The combination of root dentin formation followed by filling with bulk‐fill resin composite reduced root dentin stress.

## 1 Introduction

Traumatic dental injuries represent one of the primary causes of tooth loss and pulp necrosis in adolescents and children. Pulp necrosis in immature teeth arrests further root development, resulting in open apical foramina and large root canals [1]. Incompletely formed teeth have short and thin dentine walls, which are more prone to cervical root fracture caused by physiological biting load or secondary trauma, leading to reduced prognosis [2]. The strengthening of immature necrotic teeth represents a significant endodontic and restorative challenge.

Regenerative endodontic treatment (RET) was introduced as a treatment option and is a biologically based procedure that replaces the original pulp‐dentin complex cells with mesenchymal stem cells [3, 4]. The primary goal of RET is symptom elimination and bone healing [4]. However, root maturogenesis is an additional benefit that improves the long‐term survival of immature teeth from a biomechanical perspective [4]. The continuation of the dentin deposition promoted by RET results in thicker canal walls, increased root length and apical maturation [5].

Root development achieved by RET presents high variability, with dentin thickness increases reaching up to 70% [5]. Nevertheless, the cervical third of the root often remains weakened due to the placement of coronal plugs at the cementoenamel junction [6]. Although biological repair of the access cavity at the cervical root level with a hard tissue bridge has been reported among the favourable outcomes of regenerative endodontic procedures [7], this outcome is not an intended goal of the procedure. Several materials have been recommended for filling the cervical area of the root canal and reinforcing immature teeth [8]. Resin composite, which has a stiffness similar to root dentin, inserted into the root canal may increase the mechanical resistance of the roots through bonding interactions with the dentin walls [9]. Dentin is a hard but elastic tissue, with high deformation capacity [10]. This tissue functions physiologically to accommodate chewing forces and distribute resultant stresses evenly to adjacent structures, minimising the stress concentration [10]. Therefore, a material with elastic modulus similar to dentin, such as resin composite, can mimic the root dentin, reducing stress concentrations and producing better biomechanical results in endodontically treated teeth [9].

Overall success rates of endodontic regeneration procedures range from 50% to 98%, with survival rates between 94% and 100% [11]. Despite these promising outcomes, there is a limited number of studies assessing the biomechanical performance of RET in relation to root development. In theory, teeth treated with RET should be stronger compared to other therapeutic approaches for necrotic immature permanent teeth [12]. However, this has yet to be proven. This study aimed to investigate the stress distribution under a biting load on teeth treated with RET with different root development outcomes, compared with a tooth treated with MTA apexification reinforced with intra‐radicular resin composite, using two‐dimensional finite element analysis (2D FEA). The null hypothesis was that neither MTA apexification reinforced with intra‐radicular resin composite nor the increase in root length and thickness promoted by RET would affect the stress distribution in immature permanent traumatised teeth.

## 2 Materials and Methods

**FIGURE 1:**
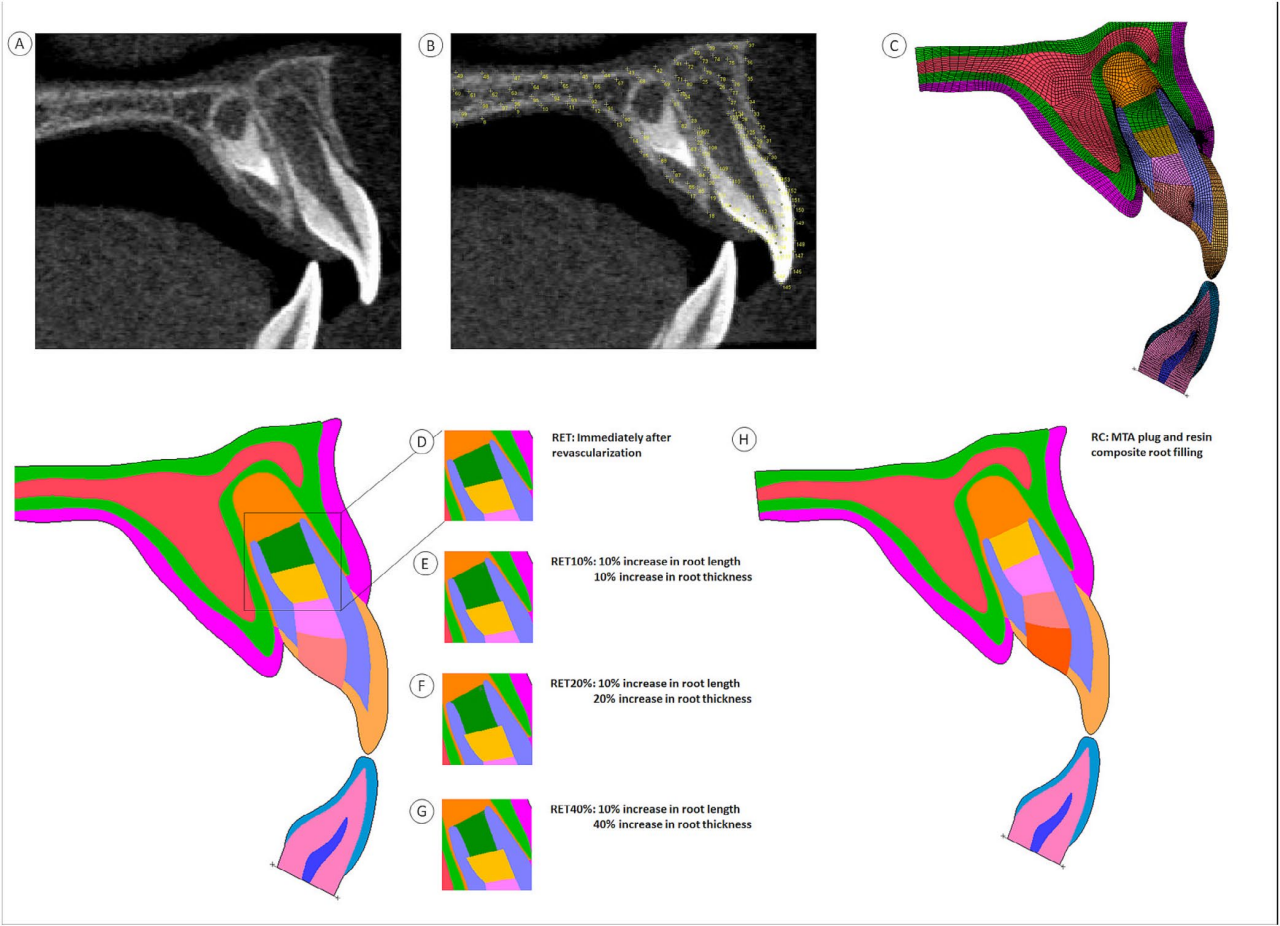
Generation of cross‐sectional finite element models. (A) CT‐tomography image of an immature maxillary central incisor; (B) Points traced on the CT image in an image processing software; (C) Mesh of the traumatized tooth and antagonist model; (D) RET‐immediately after RET; (E) RET10%, 10% increase in root length and thickness after RET; (F) RET20%, 10% increase in root length and 20% increase in root thickness after RET; (G) RET40%, 10% increase in root length and 40% increase in root thickness after RET; (H) RC‐3 mm apical MTA plug following filling of the root canal with a bulk‐fill resin composite.

This study was approved by the local ethics committee (CAAE: 05397412.0.0000.5152). Images from a cone‐beam computed tomography (CBCT) scan of a child aged 7 years old from the image bank of the School of Dentistry, Federal University of Uberlândia, Uberlândia, MG, Brazil (Figure 1A), were used to create two‐dimensional (2D) models. An image of the sagittal cross‐section of the maxilla and mandible was exported to an image processing software program (Image J, Public domain, National Institute of Health, Bethesda, MD, USA). Points were recorded on the outlines of cortical and trabecular bone, enamel, dentin, pulp, periodontal ligament, and surrounding soft tissue using the multi‐point tools (Figure 1B). The coordinates obtained were exported to a FEA software package (Marc/Mentat, version 2010.2, MSC software, Santa Ana, CA, USA) and cubic–spline curves were drawn through them, recreating the tissue outlines for the FEA. The finite element mesh consisted of solid‐shell elements of types 114 and 115. Interface elements 114 and 115 were employed to simulate distinct mechanical behaviours: element 114 modelled frictional contact without adhesion between surfaces. In contrast, element 115 represented cohesive interfaces with potential damage, simulating adhesive failure or delamination under functional loading. Following a 5% convergence analysis [13], the mesh resolution was defined. The minimum edge length was 0.0148 mm (14.8 μm), corresponding to the smallest distance between adjacent nodes in the most refined regions of interest. The maximum edge length in less critical areas was 1.139 mm. This configuration ensured a locally refined mesh in areas with high‐stress gradients while maintaining computational efficiency in less critical regions (Figure 1C). Five FEA models simulating different treatment scenarios of an immature maxillary central incisor with thin dentin walls, a wide root canal, and a divergent apical opening at stage 3 of Cvek's root development classification [1] were created as follows:

RET: post‐operative scenario following placement of a 3 mm mineral trioxide aggregate (MTA) plug at the cervical root level, without any additional increase in root structure (Figure 1D).

RET10%, post‐operative scenario with 10% increase in root length and thickness using a 3 mm coronal MTA plug in the root canal following RET (Figure 1E).

RET20%, post‐operative scenario with a 10% increase in root length and a 20% increase in root thickness using a 3 mm coronal MTA plug in the root canal following RET (Figure 1F).

RET40%, post‐operative scenario with 10% increase in root length and 40% increase in root thickness using a 3 mm coronal MTA plug in the root canal following RET (Figure 1G).

RC, immediate post‐operative scenario using a 3 mm apical MTA plug following filling of the root canal with a bulk‐fill resin composite (Figure 1H).

All interfaces were considered bonded contact preventing relative motion along all model interfaces. Nonbonded contact was prescribed between the antagonist tooth and the maxillary incisor. The models were submitted to a 100 N loading, simulating a bite through the antagonist contact envelope movement between the maxillary and mandibular incisors. The functional contact loading was simulated in 50 increments, with 2 N of load for each increment. A nodal displacement constraint (model fixation) was applied on top of the bone structure on the x‐ (horizontal) and y‐ (vertical) axes. A nonlinear structural analysis was performed the materials were considered linear, isotropic and homogeneous. The mechanical properties required for FEA expressed by elastic modulus (E) and Poisson's ratio (ʋ), and the compressive strength (CS) and diametral tensile strength (DTS) for all structures necessary for modified Von Mises stress calculation are shown in Table 1 [14, 15, 16, 17, 18, 19, 20]. The elastic modulus of newly formed dentin resulting from RET was adjusted to reflect changes associated with reparative dentin [16]. The stress distributions and residual shrinkage stress in all models were analysed using modified von Mises equivalent stress (MVM). The modified von Mises criterion incorporates differences in compressive and tensile strengths in the classic von Mises criterion by including the hydrostatic stress (I_1_) [15]. The expression for MVM stress becomes: $\sigma_{\mathrm{mvm}}=\frac{\left(\mathrm{SDE}-1\right)I_{1}+\sqrt{{\left(\mathrm{SDE}-1\right)}^{2}I_{1}2+12\mathrm{SDE}J_{2}}}{2\mathrm{SDE}}$ [15], where I_1_ is the first invariant of stress tensor: I_1_ = (σ_1_ + σ_2_ + σ_3_) and J_2_ is the second invariant of the deviatoric stress tensor: J_2_ = [(σ_1_ − σ_2_)^2^ + (σ_2_ − σ_3_)^2^ + (σ_3_ − σ_1_)^2^]/6, where σ_1_, σ_2_ and σ_3_ are the first, second and third principal stress, respectively. The mean values of the 10% highest MVM stresses were determined for the enamel and dentin at shrinkage and at the final loading application. The modified von Mises stresses values were analysed qualitatively. The stress values are expressed using a linear colour scale bar, where yellow and light grey represent the highest stress values and blue and dark grey represent the lowest stress values. The ratio between the highest MVM stress value concentrated in dentin at the final loading application and dentin area for each model was determined. The dentin area was calculated with the aid of an image processing software program (Image J) through the region of interest manager.

**TABLE 1:** Mechanical properties for dental structures, MTA and resin composite applied in FEA.

Materials/structures	Elastic Modulus (MPa)	Poisson's ratio	Diametral tensile strength (MPa)	Compressive strength (Mpa)	References
Enamel	84 100	0.30	10.3	384.0	Vilela et al. [14]; Versluis and Tantbirojn [15]
Dentin	21 960	0.31	98.7	297.0	Wang et al. [16]; Versluis and Tantbirojn [15]
Secondary dentin	20 800	0.31	98.7	297.0	Wang et al. [16]; Versluis and Tantbirojn [15]
Pulp	2	0.45	2.94	2.94	Tanaka et al. [17]
Periodontal ligament	50	0.45	—	—	Vilela et al. [14]
Cortical bone	13 700	0.33	—	—	Vilela et al. [14]
Trabecular bone	1400	0.31	—	—	Bragança et al. [18]
Soft Tissue	1.80	0.30	—	—	Bragança et al. [18]
MTA	11 760	0.31	10.5	53.5	Belli et al. [19]
Tetric N‐Ceram	12 300	0.24	39.5	224.1	Borges et al. [20]

## 3 Results

**FIGURE 2:**
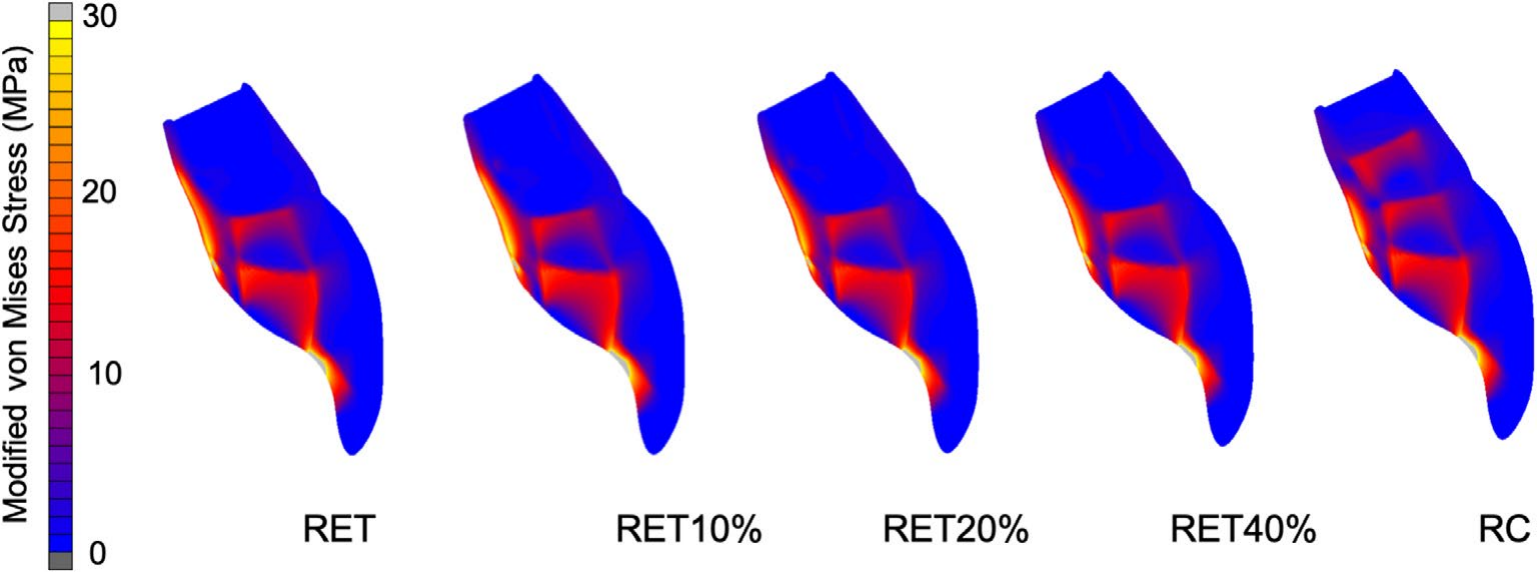
Shrinkage stress (MPa) distribution for RET, immediately after RET; RET10%, 10% increase in root length and thickness after RET; RET20%, 10% increase in root length and 20% increase in root thickness after RET; RET40%, 10% increase in root length and 40% increase in root thickness after RET; RC, 3 mm apical MTA plug following filling of the root canal with a bulk‐fill resin composite.

Modified von Mises shrinkage stress generated for all models simulating RET and MTA apexification resin composite restored model is shown in Table 2 and Figure 2. All models showed high shrinkage stress concentrations at the increment interfaces (Figure 2).

**TABLE 2:** Mean and standard deviation of top 10% stresses (MPa) on enamel and dentin at shrinkage stress.

Groups	Enamel	Dentin
RET	18.6 (4.3)	5.1 (1.5)
RET10%	18.6 (4.3)	5.1 (1.5)
RET20%	18.6 (4.3)	5.1 (1.5)
RET40%	18.9 (4.3)	5.1 (1.5)
RC	19.4 (5.1)	7.2 (1.9)

The mean of the 10% highest modified von Mises stress values (MPa) in enamel and dentin at the peak of final biting loading is shown in Table 3. Higher stress values in dentin and enamel were observed for models with newly formed dentin (RET10%, RET20% and RET40%) compared with the model simulating the immediate preoperative of the RET (RET). The RC model had the lowest dentin stress values.

**TABLE 3:** Mean and standard deviation of top 10% stresses (MPa) on enamel and dentin at a final movement of FEA.

Groups	Enamel	Dentin
RET	22.0 (8.7)	23.3 (4.4)
RET10%	22.0 (9.1)	24.8 (4.6)
RET20%	21.6 (8.6)	22.4 (4.2)
RET40%	21.6 (8.6)	20.1 (4.3)
RC	22.6 (8.7)	16.1 (7.3)

**FIGURE 3:**
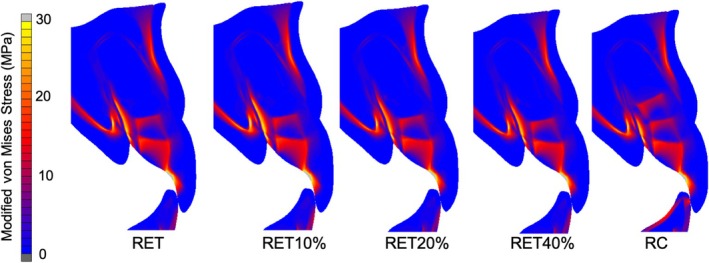
Modified Von Mises stress distributions at final movement after applying a 100 N force mimicking bite loading. RET, immediately after RET; RET10%, 10% increase in root length and thickness after RET; RET20%, 10% increase in root length and 20% increase in root thickness after RET; RET40%, 10% increase in root length and 40% increase in root thickness after RET; RC, 3 mm apical MTA plug following filling of the root canal with a bulk‐fill resin composite.

Modified von Mises equivalent stress distributions for the different models are shown in Figure 3. The models RET10%, RET20% and RET40% showed the highest stress concentrations at the cervical region, especially in the palatal face. For the model simulating the complete filling of the root canal with bulk‐fill resin composite (RC) lower stress concentration was observed at the cervical region, and more homogeneous stress distribution at root dentin compared to the other models was observed.

The ratio between the highest modified von Mises equivalent stress value in dentin at the final loading application and dentin area to each model is shown in Table 4. Higher values were demonstrated for RET compared with RET10%, RET20% and mainly RET40%. The RC model had intermediate values.

**TABLE 4:** Ratio between the highest MVM stress value in dentin (MPa) at a final movement of FEA and dentin area (mm^2^).

Groups	Area (mm^2^)	Highest MVM stress value (MPa)	Highest MVM stress value/area (MPa/mm^2^)
RET	35.9	33.5	0.93
RET10%	37.9	35.6	0.94
RET20%	39.0	32.7	0.83
RET40%	39.8	32.7	0.82
RC	35.9	32.5	0.90

## 4 Discussion

The null hypothesis was accepted since the different outcomes in RETs promoted no significant variation in the stress distribution in dentin compared to the immediate postoperative scenario. Although it was expected that the root development would reduce the stress values, as previously reported [4, 6] the deposition of mineralised tissue led to a slight increase in stress values after RET, regardless of the level of root development. The rate of root maturogenesis after RETs is variable due to individual parameters such as patient age, apical diameter, and stage of root development, which could influence the degree of root development after RET [5]. A prospective study demonstrated that root length can vary from 2.7% to 25.3% and root dentin thickness from 1.9% to 72.6% [5]. Considering this high variability, the present study fixed the increase in root length at 10%, while varying the root dentin wall thickness from 10% to 40%. An increase of 20% in root dentin thickness could be considered clinically meaningful [20, 21]. The results of the current study do not support that assumption, since our results suggest that in severely immature teeth, even a 40% increase in dentin thickness did not significantly improve the stress distribution in the dentin.

Greater predisposition to fracture in traumatised teeth according to the stage of root development has been reported [1]. In the present study, stress distribution was similar for all RET models. The similarity between the simulated conditions is supported by a recent study that demonstrated a similar fracture resistance and failure pattern for teeth treated with RET in both early and late stages [12]. The root tip at Stage 3 on Cvek's classification could provide biomechanical behaviour similar to that of mature teeth [12]. Although the methodologies are not the same, analysis of stress distribution by FEA complements the information from mechanical tests, such as fracture resistance, and explains the failure patterns [9].

In this study, a 100 N functional biting load was used, based on values from previous studies which were found to vary between 59.5 N and 155 N [22, 23]. In order to mimic the antagonist contact, envelop movement that occurs in the anterior bite, the lower incisor touches the upper incisor and slides backwards until it reaches the palatal middle third of the crown. To calculate the stress distribution RE on experimental models, modified Von Mises stress failure criterion was used. Stress distribution pattern analysis in dentin showed that the cervical third of the root developed peak stresses in all the models. This area is, therefore, more prone to the development of fracture lines on loading and agrees with other FEA studies [6, 9, 24]. This may be because of the cervical constriction and also due to the cervical region acting as a fulcrum on masticatory load application, leading to stress concentration [3]. At physiological biting, the crown tends to move in the buccal direction while the root apex moves in the palatal direction. Since the palatal cortical bone surfaces surrounding the tooth move away from the rotation center, stress is expected to increase in the palatal cervical area with the force absorbed by the periodontal ligament [3].

Endodontic access cavities in immature teeth can result in a wide access cavity to gain access to the wide canal. Usually, this access cavity is restored at the cervical level of the root with biocompatible/bioactive calcium silicate plugs combined with a composite resin restoration, which hinders the reinforcement of the immature tooth at the cervical level [6]. Curiously, most of the regenerative literature focused on the continuous root development and the dentin wall thickening potential, and little attention was given to the cervical area. The biological repair of the cervical region is as important as continuous root development and dentin wall thickening for the long‐term prognosis of regenerative endodontic procedures and might account for the true fortification of the necrotic immature teeth [7]. In the present study, the similar behaviour between RET groups regarding the stress distribution pattern is justified since all groups remain with thin and fragile cervical root dentin.

Despite the limited biomechanical reinforcement observed in the cervical region, it is important to recognise that regenerative endodontic therapy offers significant biological benefits that extend beyond structural outcomes. RET promotes the resolution of periapical infection, potential reestablishment of pulp vitality, and continued root development in immature teeth [5, 10]. Moreover, the newly formed tissues may be able to perceive external stimuli and mount an immuno‐inflammatory defence mechanism against foreign invaders, further enhancing the biological resilience of the treated tooth [25]. These aspects are particularly valuable in paediatric patients, as they contribute to the long‐term preservation of the tooth and may reduce the need for future complex interventions. Although not addressed in detail in the present study due to its biomechanical focus, such biological advantages must be considered when selecting the most appropriate treatment strategy. In vitro studies have suggested a core built‐up using resin composite into the root canal aiming to reinforce the immature teeth by bonding interaction to root dentin mediated by adhesive procedures [9]. The conventional resin composite can have a limited polymerisation at the deeper area of the root canal due to light attenuation caused by the distance from the light curing unit tip. Therefore, a bulk‐fill composite resin was chosen to restore the MTA treated immature anterior teeth. Bulk‐fill resin composites allow application of increments from 4 to 5 mm thickness, with a high degree of conversion associated with reduced shrinkage stress [26]. Shrinkage stress generated during the resin composite bonded to dentin surface may result in crack propagation, marginal loss, secondary caries and postoperative sensitivity in vital teeth [26]. Inside the root canal, high shrinkage stress can cause debonding at the adhesive/resin composite/dentin interfaces [9]. Since shrinkage stresses cannot be measured directly, the finite element analysis (FEA) is considered the most comprehensive computational method to calculate the complex stress condition within materials and dental structures [27, 28]. The RC group presented the highest shrinkage stress value due to the presence of additional resin composite increment for filling the root canal, supporting the theory that a higher number of increments can increase shrinkage stresses [28]. Shrinkage stress is a greater concern immediately after the restorative procedure because it can cause postoperative sensitivity and interface failure [29]. However, the stress levels at the interfaces were always lower than the bonding strength achieved with adhesive procedures. On the other hand, this group showed the lowest values of stresses in the dentin during functional loading, which may be related to the similar elastic modulus between bulk‐fill resin composite and dentin. Elastic modulus is defined as the ratio of stress to the corresponding strain in a material under loading [19]. The resin composite elastic modulus influences the residual stress distribution in the remaining tooth structures and at the tooth/restoration interfaces during the loading [9]. The similar stiffness between the resin composite and root dentin associated with adequate bonding interface can create a unified body that facilitates stress dissipation, thereby reducing the stress concentration [19].

The present study demonstrated that the deposition of mineralised tissue after RET increased stress. Despite that, the increase in root dentin distributes the stresses over a larger area, as shown in the ratio between the highest modified von Mises equivalent stress value and dentin area. This is related to the fact that the new‐formed tissue has a higher modulus of elasticity compared to the pulp. Dentin itself is a hard but elastic tissue and has the physiological function to bear chewing forces and distribute them equally to minimise stress [10]. In this sense, studies have suggested that the deposition of tubular dentin might also have biomechanical advantages over reparative tissue due to a broader stress distribution along the root [4]. Furthermore, according to the stress distribution pattern, the highest stresses were concentrated in the superficial dentin. Dentin becomes weaker closer to the pulp than near the dentin enamel junction, with average values of ultimate tensile strength of 61 MPa for superficial dentin and 33 MPa for deep dentin [26]. The maximum stress values found in the present study were lower than the ultimate tensile strength values, suggesting that they would not be sufficient to cause failure.

Few studies in the literature have evaluated the stress distribution in teeth that have undergone RET [4, 6], which demonstrated reduced stress by using finite element simulation. However, in these studies, immature teeth with a more advanced root development stage were simulated compared to the present study. The analyses were performed for Von Mises equivalent stresses using a three‐dimensional (3D) FEA [30]. While the equivalent Von Mises stresses integrate all stress components into an equivalent stress value, the modified von Mises criterion is interesting because it considers the ratio between compressive and tensile strength, providing greater emphasis on regions that are more likely to fail under tension in fragile structures like dentin. Considering the averages of the 10% highest modified von Mises stresses for each structure eliminates the influence of singular peak values on the results, which would not happen when calculating the maximum principal stress. In addition, the loading scenario evaluated was different, being applied to a traumatic horizontal force load of 400 N [6] or a biting scenario with a load of 240 N in the incisal edge with an angle of 120° to the long axis of the tooth and a traumatic horizontal force load of 300 N [4]. Considering that the force impacts stress distribution, these studies cannot be directly compared to what is being presented in the current study.

A finite element analysis was performed using a two‐dimensional model rather than a three‐dimensional model. When the focus is on qualitative analysis, 2D models can provide comparable results in terms of stress distribution patterns, although the stress magnitudes differ significantly [31]. The 3D models can better reproduce clinical characteristics; however, in some cases, this model can hinder the obtaining of a fine mesh, especially in thin regions, which can produce highly distorted elements and unreliable numerical results. Moreover, there are studies validating the use of 2D models to assess different conditions in anterior teeth in the cross‐sectional plane [14, 18]. The models outlined in the present study followed the same principles previously proposed [14, 18]; however, the referred studies evaluated the distribution of impact stresses during dental trauma, and the present work evaluated a physiological loading scenario.

The finite element results indicated that the stress distribution was similar qualitatively in all models but the stress magnitude was quite different. It was concluded that 2D models are acceptable when investigating the biomechanical behaviour of upper central incisors qualitatively. However, quantitative stress analysis is less reliable in 2D FEA analysis. The 2D models overestimate the results and do not represent the complex anatomical configuration of dental structures [29]. The results of the present study can be influenced by this simplification and the 3D reconstruction should be encouraged to confirm these findings with 2D models [30].

Despite the limitations of this study, since FEA presents idealised and simplified assumptions, in cases where it is not possible to test real situations in vivo or in laboratory conditions, this method has been shown to be a useful tool [3, 19]. A single model is not a realistic parameter for extrapolating results to the general population [22]. This generalisation represents a common limitation in FEA studies. Future studies should consider this aspect by creating at least three to five different specific human models to improve the generalisability of the results [22]. Another limitation of this study is the lack of validation of the FEA models. However, validation in FEA involving dental trauma is particularly challenging. Future studies aiming to develop experimental models and perform laboratory validation represent an important opportunity for further research. This limitation was addressed in the discussion section [3]. Based on the current results, it is suggested that MTA apexification followed by tooth filling using bulk‐fill resin composite is promising for the management of immature non‐vital maxillary incisors. Since there was not lower stress shown cervically in RET, this treatment modality did not have significant advantages for its recommendation.

A further limitation of this study is that RET protocols are a process of repair rather than true regeneration [33]. These authors, in an immunofluorescence study of a human teeth, reported that the neo‐mineralised tissue exhibited more collagen and lower mineral content that did not interlock into the dentinal walls. However, it was suggested that the locking projections may lack strength as the newly formed tissue was shown to easily dissociate from the canal walls. Therefore, the results of the current study for RET may be overstated as models assumed dentin regeneration rather than repair.

The results presented should be interpreted with caution, and clinical decisions regarding treatment options for immature teeth should also consider factors such as the stage of root development and the patient's preferences. Both the treatment of immature teeth with pulp necrosis using an MTA apical plug and RET result in high survival and success rates [32]. However, the existing literature lacks high‐quality clinical studies directly comparing these two treatment modalities. Further scientific investigations should focus on conducting long‐term retrospective clinical studies with rigorous methodologies to provide robust and comprehensive data supporting the biomechanical and clinical performance of these treatments over time.

Moreover, the present experiment focused on static loads, which do not accurately represent clinical conditions. It is well understood that teeth are subjected to repetitive loading, where fatigue is likely the primary cause of failure rather than exceeding a single maximum load. Future studies involving the use of the extended finite element method (XFEM) could explore crack propagation under dynamic loading [34], providing a better reflection of the clinical scenario. Within the limitations of this study, it can be concluded that the increase in root dentin thickness after RET had a limited benefit on the stress distribution pattern of immature permanent teeth under physiological loading. The combination of root dentin formation stimulation followed by tooth filling using bulk‐fill resin composite reduced root dentin stress at the cervical region.

## Author Contributions

G.L.S., B.K., C.J.S. and C.C.G.M. designed the research study. G.L.S., G.F.B., A.K.A.R., A.B.F.V. performed the research. G.L.S., A.K.A.R., and G.F.B. analysed the data. G.L.S., C.J.S. and C.C.G.M. wrote the paper. All authors have read and approved the final manuscript.

## Ethics Statement

This study was approved by the local ethics committee (CAAE: 05397412.0.0000.5152).

## Conflicts of Interest

The authors declare no conflicts of interest.

## Data Availability

The data that support the findings of this study are available on request from the corresponding author. The data are not publicly available due to privacy or ethical restrictions.
